# Transformation of a Benign-Appearing Fibroepithelial Lesion to a Giant Malignant Phyllodes Tumor of the Breast

**DOI:** 10.7759/cureus.32881

**Published:** 2022-12-23

**Authors:** Eve G Paxton, Helen Mabry, Lubna Alattia

**Affiliations:** 1 General Surgery, Beaumont Hospital Dearborn, Dearborn, USA; 2 Breast Surgery, Beaumont Hospital Trenton, Trenton, USA; 3 Pathology, Beaumont Hospital Trenton, Trenton, USA

**Keywords:** fibroepithelial lesion, fibroepithelial, fibroadenoma, management of phyllodes tumor, phyllodes adjuvant, giant phyllodes tumour, malignant phyllodes, phyllodes

## Abstract

This is a case of a woman who presented with a left breast mass that was initially diagnosed as fibroadenoma on core biopsy and, after three years without any surgical intervention, was found to be a malignant phyllodes tumor. Initially, a core needle biopsy of the mass showed probable fibroadenoma. Because of the initial benign seeming diagnosis and the need to treat her tongue cancer, the patient did not recognize the need for a recommended surgical consultation and excision. Three years later, she presented after the mass had enlarged to encompass nearly the whole left breast. Core needle biopsy revealed spindle cell proliferation with scattered benign-looking tubules. Due to the large size of the mass, she underwent a total mastectomy, and the final pathology demonstrated a malignant phyllodes tumor. This case demonstrates a case of progression of a benign-appearing fibroepithelial lesion to a malignant phyllodes tumor three years later.

## Introduction

Fibroepithelial lesions of the breast (FEL), including phyllodes tumors (PT) and fibroadenomas, are characterized by the proliferation of both epithelial and stromal components. The name phyllodes is derived from the Greek word for leaf. The tumor was first described by Muller in 1838. PTs account for only 0.3 to 1% of all primary breast tumors and 2.5% of all fibroepithelial breast tumors. The median age of presentation is 45 years old. Risk factors for PT are not well understood, but there is an increased risk of PTs in east Asian and Latin women. PTs are classified as benign, borderline, and malignant. The average size of a PT is 4 cm, but tumors measuring up to 40 cm have been reported in the literature [1]. Tumors larger than 10 cm are arbitrarily called giant tumors, and these are found in 20% of PTs [2]. Benign PTs are more frequent; they compose between 35% and 64% of cases, borderline PTs between 7% and 40% of cases, and malignant PTs compose 30% of cases [2,3]. Malignant Phyllodes Tumors (MPTs) are characterized by marked stromal cellularity and atypia, infiltrative margins, high mitotic rate (more than 10 mitoses per 10 high-power fields), and the presence of stromal overgrowth. Stromal overgrowth is associated with aggressive or metastatic behavior. MPTs of the breast have a higher chance of local recurrence [4]. The initial assessment of a PT consists of imaging, core needle biopsy, and physical examination. Core needle biopsy is preferred to fine needle aspiration for diagnosis. 

Recommended treatment for malignant PTs includes surgical excision. The goal of the surgical excision of both borderline and malignant phyllodes is to have at least a 1 cm tumor-free margin. Based on one meta-analysis that pooled data from multiple studies, a margin greater than 1 cm does not confer any significant advantage in terms of local control, distant metastasis, or overall survival for borderline and MPTs [5]. The risk of local recurrence for MPT is 18% [6]. Margin positivity is an independent risk factor for recurrence, while negative margins of <1 cm are not [6]. A consensus regarding tumor size being linked to an increased risk of local recurrence has not been reached. In one study, large tumor size, positive margin, and stromal overgrowth were shown to have an increased risk of local recurrence [7]. Other studies have shown that tumor size is not associated with local recurrence [8]. No axillary lymph node dissection is recommended, as phyllodes tumors rarely metastasize to the lymph nodes. Most commonly, MPTs metastasize to the lung, followed by the bones, heart, and liver. Adjuvant radiation can be of benefit to both borderline and malignant tumors but not to benign tumors. Chemotherapy is only used for patients with large, high-risk, or recurrent malignant phyllodes tumors. A meta-analysis of multiple studies demonstrated that adjuvant radiation is effective in preventing metastasis and recurrence but has no effect on survival [9].

## Case presentation

**Figure 1:**
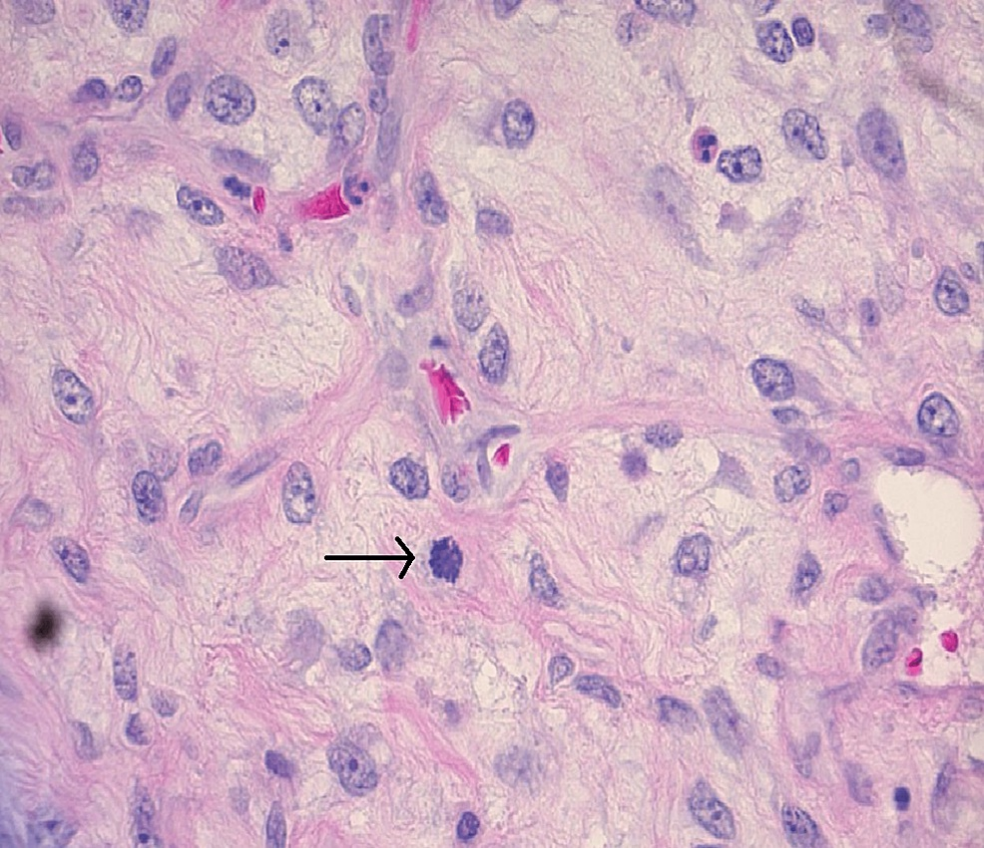
Hematoxylin and Eosin Stain, 400X magnification. One mitosis (arrow) per high-powered field from the initial biopsy

**Figure 2:**
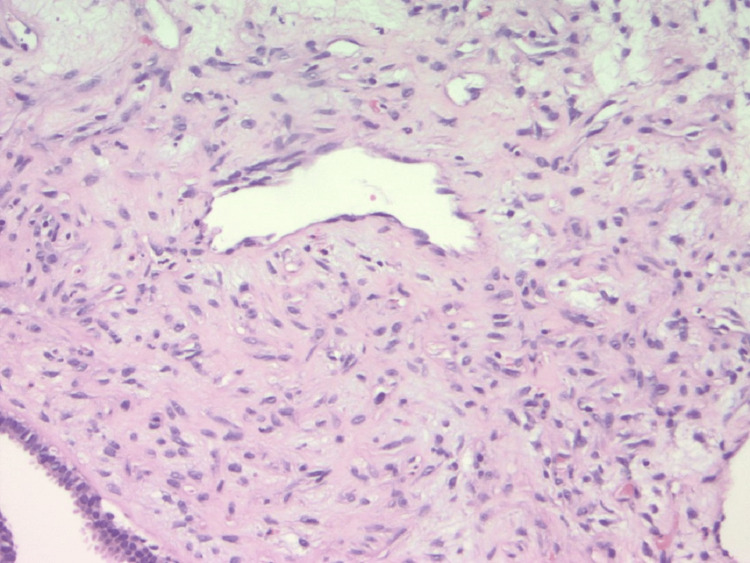
Hematoxylin and Eosin Stain, 200X magnification. Initial biopsy showed increased stromal cellularity with no heterologous elements

**Figure 3:**
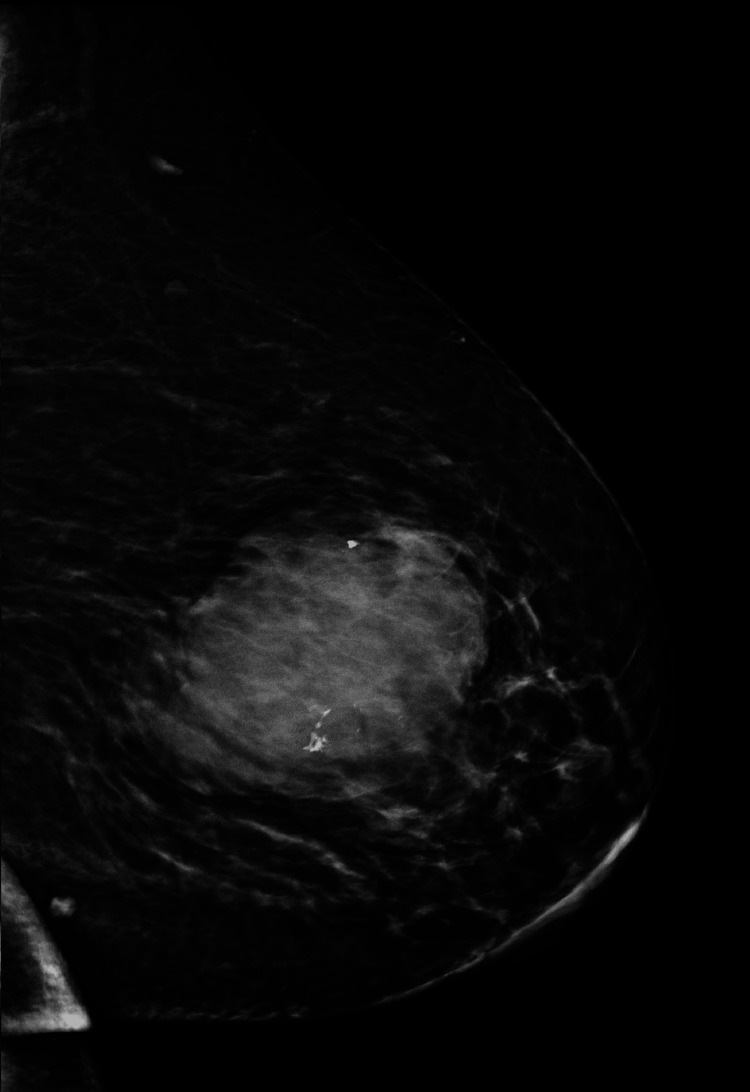
Initial left breast mass mediolateral oblique mammogram

**Figure 4:**
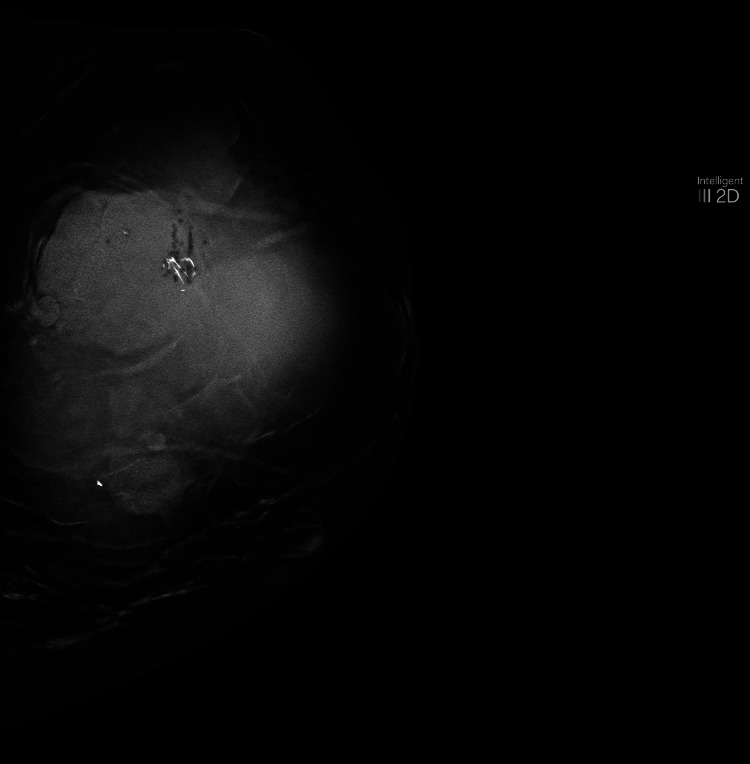
Craniocaudal mammogram of enlarged left breast mass three years after initial mammogram

**Figure 5:**
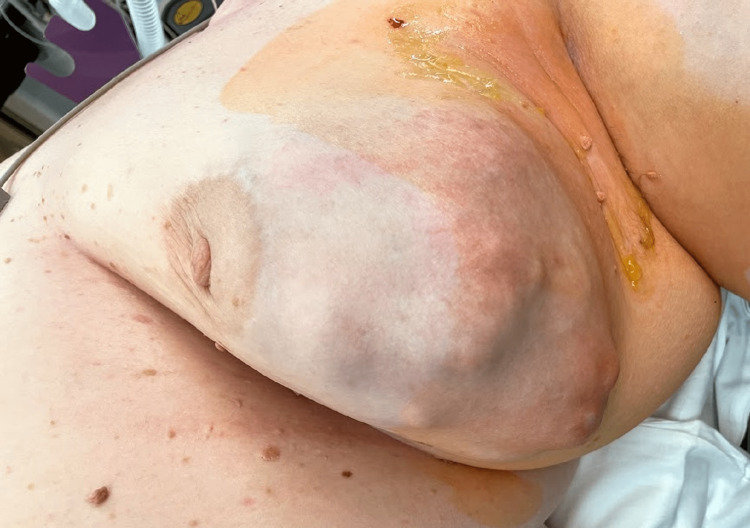
Firm, lobulated, and large left breast mass before left-sided mastectomy

The patient is a 63-year-old obese nulliparous female who presented to the clinic for evaluation of a left breast mass that had been present for two years. She underwent menopause at age 61 and menarche at age 14. She was a former smoker and smoked 6-8 cigarettes per day for 15 years. Her maternal aunt had breast cancer. Her mammogram in 2014 showed multiple bilateral breast masses, likely bilateral fibroadenomas; however, she did not have biopsies at that time. On physical exam three years before her presentation, the patient's breasts had no suspicious lesions, erythema, edema, puckering, or dimpling. The right breast had no discrete masses, but the left breast had a 6 x 6 cm mass on palpation located at the two o'clock position. Her mammogram at that time demonstrated a lobulated left breast mass measuring 6.2 x 6.5 x 6.6 cm with associated increased vascularity at the two and three o’clock positions. The lesion was classified as BIRADS 5. She had a core needle biopsy of the mass that demonstrated dense fibrous breast tissue with degenerative changes and scattered chronic inflammation, and per pathology at the initial institution where the biopsy was performed, the biopsy specimen was a probable fibroadenoma. Because the imaging and pathology were discordant, an excisional biopsy was recommended. Due to the COVID-19 pandemic and other health issues, including oral cancer, she did not follow up until three years later. Upon reassessment of the original core biopsy slides when the patient first presented, two breast pathologists diagnosed the lesion as a benign phyllodes tumor. The slides demonstrated occasional mitoses, no heterologous elements but intermediate cellularity and stromal overgrowth (Figures 1, 2). This diagnosis contradicted the initial core needle biopsy from the outside hospital. Three years later, she presented to the outpatient clinic at the secondary institution, and on the physical exam, a firm, lobulated, and large left breast mass was detected. She was reporting some left breast discomfort, but otherwise, she had no other symptoms. She underwent a diagnostic mammogram and left breast ultrasound which demonstrated a mass that measured 14.5 x 11 cm. The targeted ultrasound of the mass demonstrated a large, irregular, mixed echogenic mass with significant vascularity and benign-appearing lymph nodes in her axilla. The lesion was classified as BIRADS 4. Core biopsies contained spindle cell proliferation with scattered benign-looking tubules. The spindle cells showed mild to moderate nuclear atypia and occasional mitosis. The mammographic images of the left breast demonstrate the mass initially and the mass after it had grown for three years (Figure 3, Figure 4). The patient underwent a left-sided mastectomy (Figure 5). Pathology demonstrated an MPT. The tumor was located 8 mm from the final posterior margin. Postmastectomy, the patient, fared well, and her drain was removed on the ninth postoperative day. An oncology consultation was obtained, and a chest wall radiation course was completed, given her close posterior margin and pathological diagnosis of MPT.

## Discussion

Two possibilities can account for a core biopsy-proven fibroadenoma being rediagnosed as an MPT three years later. These include misdiagnosis at the initial core biopsy due to similar histologic appearance versus transformation of a fibroadenoma into a phyllodes tumor. The former, in this case, is more likely given the secondary review of the initial biopsy specimens, even though the transformation of a fibroadenoma into a PT can occur and has been documented in the literature [10]. 

Two elements contributed to the lesion being misdiagnosed on the initial core biopsy, which include the limitation of core biopsy in the accurate diagnosis of fibroepithelial lesions and the overlapping histological features of fibroadenoma and PT. This case highlights the well-known diagnostic dilemma of diagnosis of fibroepithelial lesions and PTs based on core needle biopsy. Core needle biopsy of PTs has a lower sensitivity of 63% based on a study comparing core needle biopsy pathology and final pathology on excision. The accuracy with which the correct fibroepithelial lesion was diagnosed on core needle biopsy was only 13% [11]. Because there is a low sensitivity, there is a higher chance of missing the diagnosis of PT on the core biopsy, as occurred with this patient. The initial core biopsy samples that were diagnosed as probable fibroadenoma were likely not from the same area that the second core biopsy samples were from that diagnosed the patient with a benign PT. Sampling error is a well-known limitation of core needle biopsy. Fibroadenomas are frequently upgraded to PTs with upstaging rates between 18% and 42% in reported case series [12]. 

Histological markers often overlap between fibroadenomas and PTs, and there are no absolute histological features to distinguish between them. Histologically, both PTs and fibroadenomas have increased stromal cellularity. Prominent mitotic activity greater or equal to 3 mitoses per 10 high power fields by itself has been shown to favor PT over fibroadenoma based on multivariate analysis [13]. There is a significant overlap in the percentage of cells staining positive for Ki-67, p53, β-catenin, and E-cadherin between low-grade PT and fibroadenoma [14]. The patient likely was initially misdiagnosed with a probable fibroadenoma on core needle biopsy when in fact, the lesion was likely a benign PT based on the secondary review of the pathology slides by additional pathologists.

The NCCN recommends excision for palpable masses suspicious for PTs that exhibit rapid growth and size greater than 3 cm on imaging, regardless of whether core needle biopsy results in a fibroadenoma or an indeterminate result. By these guidelines, this patient's breast mass would have been recommended for excision when it measured 6.6 cm on initial imaging. Based on one study for patients with nonmalignant (benign or indeterminate) and malignant PT, the overall survival was 91% and 82%, respectively, at 5 years and 79% and 42%, respectively, at 10 years [15]. If this patient would have had the lesion excised when it was initially recommended, she likely would have been able to have breast conservation, and she might have not needed radiation. Her predicted survival would also have been higher if she had it excised when initially recommended.

## Conclusions

This case highlights an important challenge for breast surgeons in the diagnosis and treatment of fibroepithelial lesions based on core needle biopsy. It supports the approach of surgical excision for fibroepithelial lesions greater than 3 cm due to the high rate of upstaging. This case is a reminder that even a benign-appearing fibroadenoma on core biopsy can have malignant potential and that the initial core needle biopsy of a fibroepithelial lesion might have lower accuracy than one might initially believe.
